# A pilot study on the neurocognitive effect of sorafenib on patients with desmoid tumours: the SORA-COG study

**DOI:** 10.3332/ecancer.2025.1871

**Published:** 2025-03-13

**Authors:** Kamboji Sharanya, Lulu Abdul khader, Fathima Shahama Oliyath Vazhayil, Ghazal Tansir, Vaishnavi Vishwas, Simran Kaur, Sameer Rastogi, Ratna Sharma

**Affiliations:** 1 All India Institute of Medical Sciences, Ansari Nagar, New Delhi 110029, India; 2 Department of Medical Oncology, BRAIRCH, All India Institute of Medical Sciences, Ansari Nagar, New Delhi 110029, India; 3 Stress and Cognitive Electroimaging Laboratory, Department of Physiology, All India Institute of Medical Sciences, Ansari Nagar, New Delhi 110029, India; a https://orcid.org/0000-0001-9091-9480

**Keywords:** neurocognition, tyrosine kinase inhibitors, sorafenib, fibromatosis, desmoid

## Abstract

**Introduction:**

Sorafenib is a multikinase tyrosine kinase inhibitor whose prolonged use may lead to cognitive impairment due to inhibition of angiogenesis. However, data on its impact on neurocognitive function in desmoid tumours (DTs) is lacking.

**Methods:**

We conducted a cross-sectional study enrolling 50 participants including 30 with DT on sorafenib (Group 1), 10 treatment-naive patients with DT (Group 2) and 10 healthy controls (Group 3). Questionnaire-based assessment was done using appearance anxiety inventory, Depression anxiety and stress questionnaire-21 (DASS-21), Hindi mental status examination (HMSE) and computer-based Cambridge neuropsychological test automated battery (CANTAB) neuropsychological tests. Statistical analysis was performed using GraphPad Prism software version 10 and individual groups were compared using Kruskal-Wallis test with *p* value of <0.05 considered significant.

**Results:**

50 participants had a median age of 29.91 years (25%–75% CI: 27.05–32.77 years), female predominance (*n* = 26, 52%), median duration of sorafenib 18.1 months (range: 6–60) at a median dose of 241.17 mg (range: 200–400). There was a statistically significant difference in anxiety (*p* = 0.0127) between Groups 1 (patients on sorafenib) and 3 (healthy controls), while other objective cognitive and neuropsychological parameters were comparable. There was a significant positive correlation between sorafenib duration and anxiety (*p* = 0.026) by DASS21, and a negative correlation between HMSE and mean five choice reaction time (*p* = 0.04).

**Conclusion:**

This is the first study to examine the neurocognitive effects of sorafenib in patients with DT. We exhibit significantly higher anxiety in patients with DT on sorafenib as compared to healthy controls. There was a significant correlation noted between anxiety and treatment duration. We propose further longitudinal studies to assess the effect of sorafenib in DT.

## Introduction

Desmoid tumour (DT) is a rare benign tumour affecting individuals aged between 25 and 40 years [1] who present with swelling, deformity, pain, loss of range of motion but may also have threatened functioning of vital organs [2]. These patients may also experience insomnia, anxiety, depression, social isolation, interruption of employment and long-term opioid use [3]. Surgical resection of DT has been found to have a recurrence rate as high as 60% [4], hence the current treatment guidelines recommend observation and medical therapies as frontline management in most cases [5].

Sorafenib, a small molecular multikinase tyrosine kinase inhibitor (TKI), is a medical therapeutic agent approved for use in DT [6]. The mechanism of action of sorafenib involves the inhibition of intracellular components of platelet derived growth factor receptor, vascular endothelial growth factor receptor (VEGFR), Raf kinase, Fms-like tyrosine kinase-3 [7]. These molecules are involved in vital processes such as neurogenesis and neuroprotection with Vascular Endothelial Growth Factor expression in the hippocampus found to have an impact on cognition and memory in rodent models [8]. In addition, angiogenic factors have been found to be involved in the pathogenesis of Alzheimer’s disease, motor neuron disease and cerebrovascular events [9]. These findings raise the possibility of TKIs such as sorafenib creating an impact on the cognitive skills of patients on long-term treatment.

There is scarce literature on the effects of prolonged administration multikinase TKIs on the cognitive functioning of patients with malignancies. The only study to examine this subject was conducted by Mulder *et al* [10] which reported a negative impact of TKIs on cognitive function among patients with metastatic renal cell carcinoma (RCC) and gastrointestinal stromal tumours (GISTs). The uniqueness of the patient population of DT lies in their younger age, usually indefinite usage of sorafenib and a long survivorship period which affects their quality of life (QoL), education and work spheres. There is a paucity of data in an indolent, non-metastatic condition such as DT on prolonged courses of sorafenib dosed at 400 mg daily. We thus conducted the first assessment of cognitive functioning in patients with DT on sorafenib therapy using pre-validated objective and subjective tools.

## Methods

### Study design

This is a cross-sectional study conducted at the Department of Medical Oncology, All India Institute of Medical Sciences, New Delhi, India. The participants included consenting individuals aged between 15 and 45 years with a convenient sample size of 50 as per feasibility. These 50 individuals included patients with histologically proven DT on sorafenib for at least 6 months (*n* = 30), patients with treatment-naive DT (*n* = 10) and healthy volunteers (*n* = 10). After obtaining ethical clearance from the Institute Review Board (IECPG/666), the enrolled participants were administered a semi-structured questionnaire comprising of details on epidemiology, demographic profile, socioeconomic status per Kuppuswamy scale [11] and details of ongoing therapy if applicable.

### Questionnaire-based assessment

The following questionnaires were administered to the participants in their language of understanding:

- Appearance anxiety inventory (AAI): The AAI is a ten-question self-reported scale that measures the cognitive and behavioural aspects of body image anxiety. This scale is useful as a part of diagnosis and symptom monitoring for the management of body dysmorphia disorder. The total score of AAI ranges from 0 to 40, with a cut-off score of 20 indicative of a high risk of clinically relevant appearance anxiety [12].
- Depression anxiety and stress questionnaire (DASS 21): The DASS 21 consists of three self-reported scales designed to measure the negative emotional states of depression, anxiety and stress. The ‘Depression’ scale assesses dysphoria, hopelessness, devaluation of life, self-depreciation, lack of interest/involvement, anhedonia and inertia. The ‘Anxiety’ scale assesses autonomic arousal, skeletal muscle effects, situational anxiety and subjective experience of anxious affect. The ‘Stress’ scale assesses difficulty in relaxation, nervous arousal, being easily upset/agitated, irritable/overreactive and impatient. The subjects are asked to use four-point severity/frequency scales to rate the extent of experiencing each state over the past 1 week. The total scores (0–56) are calculated for each negative state by summation of the individual scores [13].
- Hindi mental status examination (HMSE): HMSE is a translated and adapted form of the Mini-mental status examination used for global cognitive abilities of the Hindi-speaking adult population [14]. The HMSE is a non-copyrighted, free-to-use questionnaire and is better suited for the Hindi-speaking population of India. The questionnaire has a high sensitivity (0.81) and specificity (0.60) and it allocates points for correct answers. The maximum score obtained in this questionnaire is 31 and a score of 23 is used as a cut-off for cognitive dysfunction or dementia [15].

### Neuropsychological assessment

**Figure 1.**
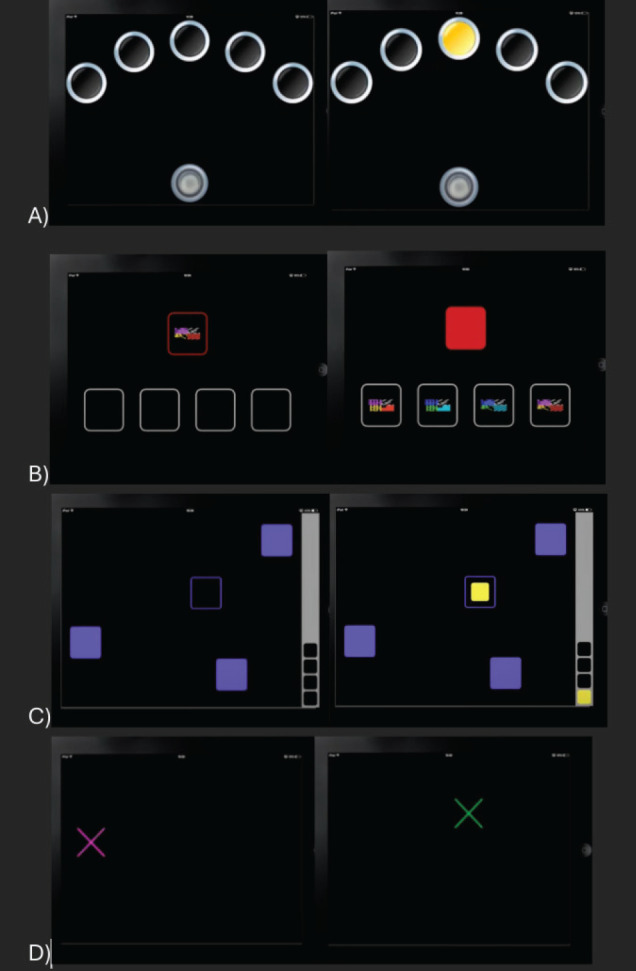
Schematic representation of the trial structures of cognitive function test battery used for objective neurocognitive assessment. (a): Delayed matching sample test. (b): Motor screening task. (c): Reaction time test. (d): Spatial working memory test.

Comprehensive computer-based cognitive function tests using Cambridge neuropsychological test automated battery (CANTAB) were applied with task-specific instructions given to the participants in the language they understood along with practice trials [16]. These tests have previously been validated for the assessment of cancer-related cognitive impairment (CRCI) among patients undergoing chemotherapy [17]. The CANTAB tests were performed in iPad with a prior explanation about the technique of the test and a trial run to ensure proper understanding. The following cognitive domains were tested using the tools mentioned below and a schematic representation is provided in Figure 1A–D.

- Processing and psychomotor speed-reaction time test: The participant selected and held a button at the bottom of the screen. Circles were presented above (one for the simple mode, and five for the five-choice mode). In each case, a yellow dot appeared in one of the circles, and the participant had to react as soon as possible, releasing the button at the bottom of the screen and selecting the circle in which the dot appeared. Outcome measures were divided into reaction time and movement time for five-choice variants and mean reaction time and error score for simple choice variant (Figure 1A). Administration time: 3 minutes.
- Attention and short-term visual memory-Delayed matching to sample test: The participant was shown a complex visual pattern, both abstract and non-verbal (the sample), followed by four similar patterns, after a brief delay. The participant had to select the pattern which exactly matched the sample. In some trials the sample and the choice patterns were shown simultaneously, in others there was a delay (of 0, 4 or 12 seconds) before the four choices appeared. Outcome measures included mean latency (the participant’s speed of response), total correct patterns selected, total errors selected and percent of correct patterns selected (Figure 1B). Administration time: 7 minutes.
- Working memory and strategy-spatial working memory test: The test began with a number of colored squares (boxes) shown on the screen. The aim of this test was that by selecting the boxes and using a process of elimination, the participant should find one yellow ‘token’ in each of a number of boxes and use it to fill up an empty column on the right-hand side of the screen. Depending on the difficulty level used for this test, the number of boxes were gradually increased until a maximum of 12 boxes were shown for the participants to search. The colour and position of the boxes used were changed from trial to trial to discourage the use of stereotyped search strategies. Outcome measures included errors (selecting boxes that have already been found to be empty and revisiting boxes that have already been found to contain a token) both within errors and total errors (Figure 1C). Administration time: 4–6 minutes.
- Sensorimotor function and comprehension-Motor screening task test: Colored crosses were presented in different locations on the screen, one at a time. The participant selected the cross on the screen as quickly and accurately as possible. Outcome measures covered mean latency, mean latency standard deviation and total correct (Figure 1D). Administration time: 2 minutes.

### Statistical analysis

Descriptive statistics provided summaries of the data, including means, medians and standard deviations to characterise the baseline details of participants, neuropsychological status and objective cognitive function assessment scores. All parameters were tested for normality using the Shapiro-Wilk test and were found to be non-normal in distribution. Data from individual groups was compared using Kruskal-Wallis test and correlation testing was done to determine statistical significance with p value of <0.05 considered significant. All the statistical testing was performed using GraphPad Prism software version 10.

## Results

A total of 50 participants were enrolled in the study and all completed the administered neuropsychological tests and questionnaires.

1. Clinico-demographic data: The study population comprised 30 (60%) patients with DT on sorafenib (Group 1), 10 (20%) patient controls with DT (Group 2) and remaining healthy controls (Group 3). The overall study population had a median age of 29.91 years (25%–75% CI: 27.05–32.77 years) and a slight female predominance (*n* = 26, 52%), with group-wise demographic details are provided in Table 1. The mean dose of sorafenib being consumed at the time of study by Group 1 was 241.1 mg (25%–75% CI: 208.50–270.90 mg) over a mean duration of 18.1 months (25%–75% CI: 11.47–24.87 months). All patients had received sorafenib as the first line of medical therapy. Univariate analysis did not yield a statistically significant difference between respective groups for age (*p* = 0.413) and education status (*p* = 0.5).
2. Questionnaire assessment scores:
  - AAI: Median scores of AAI were not statistically significantly different amongst the groups and are mentioned in Table 2.
  - DASS-21: Median scores of DASS revealed a statistically significant difference in median anxiety scores between Groups 1 and 3 (*p* = 0.0127), while the difference in anxiety scores were not statistically significant between Groups 2 and 3 (*p* = 0.1971) (Table 2). In Group 1, the median DASS-21 anxiety scores did not have a statistically significant correlation with response to sorafenib (complete/partial response versus stable/progressive disease, *p* = 0.48).
  - HMSE: Median scores of subjective cognitive assessments by HMSE were not statistically significant and are mentioned in Table 2.
3. Neuropsychological cognitive assessment: The median scores of neuropsychological cognitive assessment tests are described in Table 3. No statistically significant difference was found in:
  - Processing and psychomotor speed*:* Reaction time mean five choice movement time (RTIFMMT), reaction time mean five choice reaction time (RTIFMRT), Reaction time simple error score (RTISES) and Reaction time simple mean reaction time (RTISMRT)
  - Attention and short-term visual memory: Delayed matching to sample mean correct latency (DMSML), Delayed matching to sample total correct (DMSTC), Delayed matching to sample total errors (DMSTE) and Delayed matching to sample percent corrects (DMSPCAD)
  - Working memory and strategy: Spatial working memory within errors (SWMWE), Spatial working memory total errors (SWMTEs)
  - Sensorimotor function and comprehension: Motor screening total correct (MOTTC), motor screening mean latency standard deviation (MOTSDL) and motor screening mean latency (MOTML) parameters between the tested groups.
4. Correlation analysis: The correlation between HMSE and RTIFMRT is significant (*p* = 0.042) which says there is negative correlation of scores of HMSE and RTIFMRT (*r* = −0.39). Correlation between duration of sorafenib intake and Anxiety is significant (*p* = 0.026) with a positive correlation (*r* = 0.45). The correlation between stress and dosage of sorafenib intake is statistically not significant (*p* = 0.08).

## Discussion

DT is a rare tumour with scarce data on the treatment implications experienced by patients. Ours is the first study that attempts to investigate the neurocognitive effects of sorafenib among patients with DT, which has never been explored so far. The reasons for this may be the rarity of the condition and the lack of awareness among physicians on the possible impacts of oral targeted therapies. We also included comparator population groups comprising both patients with DT who were not receiving sorafenib as well as healthy controls which further refined our findings.

We have previously published data from our institution which showed that patients with DT on sorafenib had worse QoL in terms of symptom burden and poor functioning, and poor anxiety and depression scores compared to the healthy population [18]. Similar findings were observed in our study which showed significantly higher anxiety scores in patients on sorafenib as compared to healthy controls. It is notable that response to therapy did not correlate with anxiety among patients on sorafenib. Within the constraints of a limited sample size, this finding suggests that even those patients whose disease is responding to sorafenib experienced significant anxiety compared to healthy individuals. Additionally, preliminary findings from our institution have shown that QoL parameters improved significantly after sorafenib discontinuation among patients with DT while neurocognitive functions remained stable [19].

The CANTAB is a useful tool in neurotoxicology and has been validated among patients with breast cancers and Hodgkin lymphoma to assess chemotherapy-related CRCI [17, 20]. By the use of this battery, we were able to perform language-independent, multiple-task assessments with a reduction in the practice effects in patients. The analysis of objective and subjective cognitive function tests within our study revealed no statistically significant differences among healthy control groups, diseased controls and diseased patients. These findings suggest that, within the parameters of our study, cognitive function as measured by these tests does not significantly vary across these groups. This differs from the findings of Mulder *et al* [10] who described a notable negative difference in cognitive function among patients by both subjective and objective parameters. Their study comprised of patients with metastatic RCC and GIST on sorafenib and sunitinib, along with treatment naïve patients with RCC and healthy controls. The mean age of the patients taking TKI was 60 years and sixteen neuropsychological tests were used for assessment. On the contrary, our study comprised of a younger patient subset, having an indolent borderline-malignant condition of DT, and the application of four neuropsychological tests. These factors could account for the differences noted in the outcomes between the two studies.

We recognise that the mean dosage of sorafenib being used in the patients enrolled in our study was lesser than the recommended dose of 400 mg once a day [6]. This dosage may be more suitable for the study population in terms of neurocognitive preservation, especially because of the prolonged duration of therapy. Our study thus makes important observations of neurocognitive consequences of chronic use of anti-VEGFR TKIs and opens further possibilities of dose adjustments to minimise these effects.

The negative correlation between HMSE scores and RTIFMRT scores provides valuable insights into the neurocognitive assessment tools used. High values of HMSE scores indicates good neurocognition and low scores of RTIFMRT indicates good neurocognition so it is explainable to have a negative correlation between the scores of these two. This inverse relationship underscores the validity of these assessments in evaluating cognitive health, particularly in patients undergoing treatment. The significant inverse relationship between HMSE and RTIFMRT scores supports the reliability of these tools in assessing neurocognitive health. Higher HMSE scores reflect better mental status, while lower RTIFMRT scores indicate faster and more accurate memory recall, both of which are indicators of good cognitive function. Previous studies have demonstrated a negative correlation with reaction times and general mental ability and the expected negative correlation noted in our study between these measures validates the use of these assessments in monitoring cognitive changes over time [21]. Thus, these parameters could assist in the identification of potential cognitive impairments early in the treatment process.

The second significant finding pertains to the relationship between the duration of sorafenib intake and anxiety levels. The positive correlation suggests that prolonged use of sorafenib is associated with increased anxiety. This is an important observation for clinical practice, highlighting the need for monitoring and potentially increasing anxiety symptoms in patients undergoing long-term treatment with sorafenib. The mechanism behind this correlation could be multifactorial, involving both the direct pharmacological effects of sorafenib and the psychological burden of prolonged treatment. While correlation tests were not performed between anxiety scores and burden of disease due to the small sample size, the tumour size and site distribution of DT were comparable in both patient groups. The correlation between stress levels and the dosage of sorafenib intake did not reach statistical significance (*p* = 0.08) and might imply that other factors, such as individual coping mechanisms, support systems or concurrent treatments, play more substantial roles in influencing stress levels. Similar findings were made by a previous study conducted at our center wherein the daily dosages of 400 and 200 mg did not yield a significant different in the prevalence of anxiety (8% and 7.6%, respectively) [22].

Our study does have limitations in the form of its cross-sectional design, limited sample size and fewer neuropsychological tests. Patients aged greater than 45 years were not recruited into the study, and this could have excluded patients more prone to neurocognitive side effects. However, as a single-centre study conducted with limited funding and targeting a rare disease, it offers useful insights into future research in this domain. DT as an entity is the model that can be used to study the long-term effects of TKI use on the neurocognitive profile of patients. Thus, a prospective trial with a longer follow-up period and broader age range is warranted to explore the long-term effects of sorafenib and its implication on patient care.

## Conclusion

The study provides valuable insights into the neurocognitive effects of sorafenib treatment in patients with DTs. While the findings indicate a significant increase in anxiety levels among patients receiving sorafenib compared to treatment-naïve patients, other cognitive function parameters were comparable between all groups. The elevated anxiety levels highlight the need for comprehensive psychosocial support and monitoring during sorafenib treatment. Healthcare professionals should be vigilant in assessing mental health concerns to optimise their overall well-being and treatment outcomes. As per the observations of our study, sorafenib can be prescribed to patients with DT with no significant neurocognitive decline but these findings need to be confirmed by larger prospective studies.

## List of abbreviations

AAI, Appearance anxiety inventory; CANTAB, Cambridge neuropsychological test automated battery; CRCI, Cancer-related cognitive impairment; DASS21, Depression anxiety and stress questionnaire; DMSML, Delayed matching to sample mean correct latency; DMSPCAD, Delayed matching to sample percent corrects; DMSTC, Delayed matching to sample total correct; DMSTE, Delayed matching to sample total errors; DT, Desmoid tumour; GIST, Gastrointestinal stromal tumours; HMSE, Hindi mental status examination; Mg, Milligrams; MOTML, Motor screening mean latency; MOTSDL, Motor screening mean latency standard deviation; MOTTC, Motor screening total correct; QoL, Quality of life; RCC, Renal cell carcinoma; RTIFMMT, Reaction time mean five choice movement time; RTIFMRT, Reaction time mean five choice reaction time; RTISES, Reaction time simple error score; RTISMRT, Reaction time simple mean reaction time; SWMTE, Spatial working memory total errors; SWMWE, Spatial working memory within errors; TKI, Tyrosine kinase inhibitor; VEGFR, Vascular endothelial growth factor receptor.

## Conflicts of interest

The authors have no conflicts of interest to declare.

## Funding

Undergraduate mentorship Scheme project (Reference no:F.8.48/UG-48/2023/RS), All India Institute of Medical Science, New Delhi, granted to Kamboji Sharanya, Lulu A, Fathima Shahama OV (mentees) and Dr Simran Kaur (Mentor).

## Ethical statement

Ethical clearance to conduct the study was obtained from the Institute Review Board at All India Institute of Medical Sciences, New Delhi. (IECPG/666). Informed consent was taken from all subjects prior to participation in the study.

## Author contributions

SK and SR conceptualized the study. KS, LA, FSOV, VV performed data collection, analysis, manuscript writing, literature review. GT, SR, SK, RS performed manuscript writing and proofreading. All authors reviewed the final draft of the manuscript.

## Data availability statement

Data material of the study can be provided upon reasonable request to the corresponding authors.

## Figures and Tables

**Table 1.** Description of baseline characteristics of the study groups.

Characteristics	Patients with sorafenib (*n* = 30)	Patient controls (*n* = 10)	Healthy controls (*n* = 10)
Age, years (mean)	30.58 (18–45)	31.10(21–43)	27.14 (20–45)
Male: Female	13 :17	6 :4	5 :5
Site distribution (*n*, %)			Not applicable
Abdominal (*n*, %) Extra-abdominal (*n*, %)	5 (16.6) 25 (83.4)	2 (20) 8 (80)	-
Duration of sorafenib (months)	18.17 (6–60)	_	_
Dosage (in mg)	241.17 (200–400)	_	_
Best response to sorafenib (*n*, %)	PR (12, 40) SD (18, 60)	_	_

Abbreviations: PR, Partial response; SD, Stable disease

**Table 2.** Description of scores of objective cognitive assessment tools.

	Patients with sorafenib (A)		Patient controls (B)		Healthy controls (C)		*p*-value A versus B	*p*-value A versus C	*p*-value B versus C
	Median	(25%–75%)	Median	(25%–75%)	Median	(25%–75%)			
AAI	7.00	(3.00–12.5)	6.00	(4.00–12.0)	6.00	(5.00–9.00)	>0.999	>0.999	>0.999
Depression (DASS)	4.00	(1.00–7.25)	3.50	(1.00–5.50)	1.00	(1.00–2.00)	>0.999	0.137	0.370
Anxiety (DASS)	3.00*	(1.00–5.25)	1.50	(0.75–4.50)	0.00	(0.00–1.00)	>0.999	**0.012**	0.197
Stress (DASS)	3.50	(0.00–7.00)	4.00	(0.00–8.50)	4.00	(1.00–4.00)	>0.999	>0.999	>0.999
HMSE	30.00	(29.0–30.0)	30.00	(28.75–30.0)	30.00	(30.0–30.0)	>0.999	0.456	>0.999

Abbreviations: AAI, Appearance Anxiety Inventory; DASS, Depression Anxiety and Stress Questionnaire; HMSE, Hindi Mental Status Examination Bold indicates statistically significant *p* value(<0.05)

**Table 3.** Description of scores of neuropsychological cognitive assessment tests.

	Patients with sorafenib		Patient controls		Healthy controls		*p*-value A versus B	*p*-value A versus C	*p*-value B versus C
	Median	(25%–75%)	Median	(25%–75%)	Median	(25%–75%)			
Processing and psychomotor speed									
RTIFMMT^	294.5	(230.6–395.4)	299	(233.2–384.9)	267.7	(241.7–404.6)	>0.999	>0.999	>0.999
RTIFMRT^	429.9	(404–475.9)	430.6	(413.8–444.2)	464.8	(403.1–567.0)	>0.999	0.764	0.779
RTISES^	1.000	(0.000–3.000)	1.000	(0.000–1.250)	1.000	(0.000–1.250)	>0.999	>0.999	>0.999
RTISMRT^	395.0	(349.9–426.1)	383.5	(362.8–405.5)	429.4	(372.3–553.8)	>0.999	0.1531	0.1812
Attention and short-term visual memory									
DMSML^	3437	(2,944–4530)	3539	(2,922–4795)	3464	(2,697–4403)	>0.999	>0.999	>0.999
DMSTC	16.0	(13.0–17.50)	17.0	(14.0–18.25)	17.0	(15.75–19.0)	0.932	0.436	>0.999
DMSTE^	4.000	(2.50–7.00)	3.000	(1.75–6.00)	3.000	(1.00–4.25)	0.932	0.436	>0.999
DMSPCAD	73.00	(60.0–83.5)	83.50	(69.75–88.50)	83.50	(73.00–93.00)	0.758	0.490	>0.999
Working memory and strategy									
SWMWE^	1.000	(0.00–2.00)	3.000	(0.00–4.50)	0.000	(0.00–1.00)	0.307	0.728	0.064
SWMTE^	18	(10.50–22)	15	(8.25–23.750 )	13.00	(2.00–18.00)	>0.999	0.355	0.881
Sensorimotor function and comprehension									
MOTTC	10.00	(10.0–10.0)	10.00	(10.0–10.0)	10.00	(10.0–10.0)	>0.999	>0.999	>0.999
MOTSDL^	157.6	(109.0–272.0)	138.3	(102.6–204.9)	198.5	(137.6–326.3)	>0.999	0.504	0.435
MOTML^	815.7	(713.0–1125)	799.2	(625.3–943.7)	810.6	(696.7–1015)	>0.999	>0.999	>0.999

Higher scores reflect a better performance except for parameters labelled as ^

Abbreviations: RTIFMMT, Reaction time task Mean five choice Movement time; RTIFMRT, Reaction time task Mean Five choice reaction time; RTISES, Reaction time task simple error score; RTISMRT, Reaction time task simple mean reaction time; DMSML, Delayed match to sample mean correct latency; DMSTC, Delayed match to sample total correct; DMSTE, Delayed match to sample total errors; DMSPCAD, Delayed match to sample percent corrects; SWMWE, Spatial Working memory within errors; SWMTE, Spatial Working memory total errors; MOTTC, Motor screening task total correct; MOTSDL, Motor screening task Mean Latency Standard Deviation; MOTML, Motor screening task mean latency

## References

[1] Kasper B, Ströbel P, Hohenberger P (2011). Desmoid tumors: clinical features and treatment options for advanced disease. Oncologist.

[2] Bektas M, Bell T, Khan S (2023). Desmoid tumors: a comprehensive review. Adv Ther.

[3] Husson O, Younger E, Dunlop A (2019). Desmoid fibromatosis through the patients’ eyes: time to change the focus and organisation of care?. Support Care Cancer.

[4] Janssen ML, Broekhoven DLM, Cates JMM (2017). Meta-analysis of the influence of surgical margin and adjuvant radiotherapy on local recurrence after resection of sporadic desmoid-type fibromatosis. Br J Surg.

[5] Alman B, Attia S, Baumgarten C (2020). The management of desmoid tumours: a joint global consensus-based guideline approach for adult and paediatric patients. Eur J Cancer.

[6] Gounder MM, Mahoney MR, Van Tine BA (2018). Sorafenib for advanced and refractory desmoid tumors. N Engl J Med.

[7] Gotink KJ, Verheul HMW (2009). Anti-angiogenic tyrosine kinase inhibitors: what is their mechanism of action?. Angiogenesis.

[8] Luck R, Urban S, Karakatsani A (2019). VEGF/VEGFR2 signaling regulates hippocampal axon branching during development. eLife.

[9] Greenberg DA, Jin K (2005). From angiogenesis to neuropathology. Nature.

[10] Mulder SF, Bertens D, Desar IME (2014). Impairment of cognitive functioning during sunitinib or sorafenib treatment in cancer patients: a cross sectional study. BMC Cancer.

[11] Radhakrishnan M, Nagaraja SB (2023). Modified Kuppuswamy socioeconomic scale 2023: stratification and updates. Int J Community Med Public Health.

[12] Appearance Anxiety Inventory (2021). NovoPsych.

[13] Lovibond SH, Lovibond PF (1995). Depression anxiety stress scales. PsycTESTS Dataset.

[14] Porrselvi AP, Shankar V (2017). Status of cognitive testing of adults in India. Ann Indian Acad Neurol.

[15] Ganguli M, Ratcliff G, Chandra V (1995). A Hindi version of the MMSE: the development of a cognitive screening instrument for a largely illiterate rural elderly population in india. Int J Geriatr Psychiatry.

[16] (2023). Digital Cognitive Assessments.

[17] Janelsins MC, Heckler CE, Peppone LJ (2018). Longitudinal trajectory and characterization of cancer-related cognitive impairment in a nationwide cohort study. J Clin Oncol.

[18] Garg V, Rastogi S, Kalra K (2022). Health-related quality of life (HRQoL), anxiety, and depression in patients with desmoid type fibromatosis. Support Care Cancer.

[19] Bharath B, Rastogi S, Garg V (2023). 1914MO The clinico-radiological outcome of desmoid type-fibromatosis after discontinuing the sorafenib treatment in responders: single-arm phase II clinical trial. Ann Oncol.

[20] Magyari F, Virga I, Simon Z (2022). Assessment of cognitive function in long-term hodgkin lymphoma survivors, results based on data from a major treatment center in Hungary. Support Care Cancer.

[21] Der G, Deary IJ (2017). The relationship between intelligence and reaction time varies with age: results from three representative narrow-age age cohorts at 30, 50 and 69 years. Intelligence.

[22] Garg V, Gangadharaiah BB, Rastogi S (2023). Efficacy and tolerability of sorafenib in desmoid-type fibromatosis: a need to review dose. Eur J Cancer.

